# Supplementary material for: Mechanisms of diversity maintenance in dung beetle assemblages in a heterogeneous tropical landscape
Source: PeerJ. 2020 Sep 8;8:e9860. doi: 10.7717/peerj.9860 (PMC7903913; doi:10.7717/peerj.9860)
Supplement: Supplemental Information 1 [file peerj-08-9860-s001.docx]

Landscape composition (as percentage coverage) and configuration of each window.

| **Window/locality ^a^** | **Landscape composition ^b^** | | | | **Landscape configuration** | |
| --- | --- | --- | --- | --- | --- | --- |
|  | **F** | **SF** | **P** | **SH-LD** | **Splitting index** | **Edge density (m/ha)** |
| W1/SMR | 90.38 | 9.61 | 0 | 1.37 | 1.22 | 44.99 |
| W2/SVC | 90.38 | 9.61 | 0 | 1.38 | 2.45 | 39.28 |
| W3/NSJC | 37.23 | 51.68 | 11.09 | 2.55 | 3.45 | 175.23 |
| W4/NSJC | 42.06 | 47.19 | 10.75 | 2.53 | 6.15 | 169.28 |
| W5/SJ | 36.27 | 53.24 | 10.49 | 2.31 | 6.26 | 180.37 |
| W6/TN | 5.77 | 10.71 | 83.5 | 1.74 | 1.45 | 121.57 |
| W7/TN | 0 | 10.54 | 89.45 | 1.40 | 1.25 | 78.18 |
| W8/AZ | 1.91 | 45.31 | 52.76 | 2.16 | 2.65 | 132.94 |
| **Overall** | 38 | 30 | 32 |  |  |  |

^a^ Window/locality:W: window; SMR: Sierra Monterrey; SVC: Sierra Veinte Casas; NSJC: Nuevo San Juan Chamula; SJ: San Joaquín; TN: Tierra Nueva; AZ: Armando Zebadua.

^b^ F: Tropical forest; SF: Second-growth forest; P: Pasture; SH-LD: exponential Shannon Landscape Diversity.
