# Supplementary material for: Mechanisms of diversity maintenance in dung beetle assemblages in a heterogeneous tropical landscape
Source: PeerJ. 2020 Sep 8;8:e9860. doi: 10.7717/peerj.9860 (PMC7903913; doi:10.7717/peerj.9860)
Supplement: Supplemental Information 2 — Test comparing the expected numbers of traps (left/) under the assumption that the sampling was exactly proportional to the landscape composition of each window, and the actual number of traps placed (/right). The Fisher exact test was used for the windows and the chi-square goodness-of-fit test for vegetation classes. [file peerj-08-9860-s002.docx]

## Fisher exact test and chi-square goodness-of-fit test.

Test comparing the expected numbers of traps (left/) under the assumption that the sampling was exactly proportional to the landscape composition of each window, and the actual number of traps placed (/right). The Fisher exact test was used for the windows and the chi-square goodness-of-fit test for vegetation classes.

|  | **Fisher’s exact test for windows** | | | | | | | | ***Χ*^2^ test**  **for V.C.** |
| --- | --- | --- | --- | --- | --- | --- | --- | --- | --- |
| **VC^a^** | W1 | W2 | W3 | W4 | W5 | W6 | W7 | W8 |  |
| **F** | 38/37 | 38/36 | 15/19 | 18/17 | 15/16 | 2/1 |  | 1/1 | 127/127 |
| **SF** | 4/5 | 4/6 | 22/19 | 20/18 | 22/18 | 5/7 | 4/3 | 19/16 | 100/92 |
| **P** |  |  | 5/4 | 4/7 | 5/8 | 35/34 | 38/39 | 22/25 | 109/117 |
| **P value** | 0.67 | 0.53 | 0.75 | 0.68 | 0.68 | 0.81 | 0.99 | 0.82 | ***Χ^2^*** 1.22; **P**: 0.54 |

^a^ Vegetation classes: F: Forest; SF: Second-growth forest; P: Pasture
