# Supplementary material for: Mechanisms of diversity maintenance in dung beetle assemblages in a heterogeneous tropical landscape
Source: PeerJ. 2020 Sep 8;8:e9860. doi: 10.7717/peerj.9860 (PMC7903913; doi:10.7717/peerj.9860)
Supplement: Supplemental Information 3 — The spatial independence of the response variables (0D, 1D, abundance, biomass). [file peerj-08-9860-s003.docx]

Results of the Moran I test.

|  | Species richness (**^0^*D***) | | | Exp (Shannon Diversity) (**^1^*D***) | | |
| --- | --- | --- | --- | --- | --- | --- |
| **Sampling units** | Observed | Expected | p value | Observed | Expected | p value |
| Windows | -0.17 | -0.14 | 0.82 | -0.21 | -0.14 | 0.57 |
| Forest | -0.05 | -0.17 | 0.43 | -0.17 | -0.16 | 0.96 |
| Second-growth forest | -0.24 | -0.14 | 0.42 | -0.21 | -0.14 | 0.57 |
| Pasture | -0.20 | -0.20 | 0.93 | -0.07 | -0.2 | 0.17 |
|  | **Abundance** | | | **Biomass** | | |
| **Sampling units** | Observed | Expected | p value | Observed | Expected | p value |
| Windows | -0.25 | -0.14 | 0.37 | -0.24 | -0.14 | 0.37 |
| Forest | -0.29 | -0.17 | 0.41 | -0.29 | -0.17 | 0.42 |
| Second-growth forest | 0.13 | -0.20 | 0.58 | -0.26 | -0.14 | 0.30 |
| Pasture | -0.21 | -0.2 | 0.89 | -0.16 | -0.2 | 0.74 |
