# Supplementary material for: Mechanisms of diversity maintenance in dung beetle assemblages in a heterogeneous tropical landscape
Source: PeerJ. 2020 Sep 8;8:e9860. doi: 10.7717/peerj.9860 (PMC7903913; doi:10.7717/peerj.9860)
Supplement: Supplemental Information 4 — F: Forest; SF: Second-growth forest; P: Pasture [file peerj-08-9860-s004.docx]

Species abundance and sample coverage; F: Forest; SF: Second-growth forest; P: Pasture

|  |  | **W1** | | **W2** | | **W3** | | | **W4** | | | **W5** | | | **W6** | | | **W7** | | **W8** | | | **n** |
| --- | --- | --- | --- | --- | --- | --- | --- | --- | --- | --- | --- | --- | --- | --- | --- | --- | --- | --- | --- | --- | --- | --- | --- |
| **#** |  | **F** | **SF** | **F** | **SF** | **F** | **SF** | **P** | **F** | **SF** | **P** | **F** | **SF** | **P** | **F** | **SF** | **P** | **SF** | **P** | **F** | **SF** | **P** |  |
|  | **Tribe Ateuchini Perty, 1830** |  |  |  |  |  |  |  |  |  |  |  |  |  |  |  |  |  |  |  |  |  |  |
| **1** | *Ateuchus candezei* (Harold, 1868) | 1 | 0 | 0 | 0 | 0 | 0 | 0 | 0 | 0 | 0 | 0 | 0 | 0 | 0 | 0 | 0 | 0 | 0 | 0 | 0 | 0 | 1 |
| **2** | *Ateuchus perezvelai* Kohlmann, 2000 | 1 | 0 | 0 | 0 | 0 | 0 | 0 | 1 | 0 | 0 | 1 | 0 | 0 | 0 | 0 | 0 | 0 | 0 | 0 | 0 | 0 | 3 |
| **3** | *Ateuchus rodriguezi* Preudhomme de Borre, 1886 | 4 | 0 | 0 | 0 | 6 | 12 | 0 | 31 | 0 | 0 | 3 | 9 | 0 | 0 | 0 | 0 | 0 | 0 | 0 | 0 | 0 | 65 |
| **4** | *Bdelyropsis newtoni* Howden, 1971 | 0 | 0 | 0 | 0 | 0 | 2 | 0 | 0 | 0 | 0 | 0 | 0 | 0 | 0 | 0 | 0 | 0 | 0 | 0 | 0 | 0 | 2 |
| **5** | *Scatimus ovatus* Harold, 1862 | 0 | 0 | 0 | 0 | 0 | 0 | 0 | 0 | 3 | 2 | 5 | 40 | 2 | 1 | 0 | 0 | 0 | 0 | 0 | 26 | 20 | 99 |
| **6** | *Uroxys boneti* Pereira & Halffter, 1961 | 3 | 0 | 3 | 0 | 1 | 0 | 0 | 1 | 0 | 0 | 0 | 2 | 0 | 0 | 1 | 0 | 0 | 0 | 0 | 0 | 0 | 11 |
| **7** | *Uroxys deavilai* Delgado & Kohlmann, 2007 | 0 | 0 | 0 | 0 | 0 | 0 | 0 | 0 | 0 | 0 | 0 | 0 | 0 | 0 | 0 | 0 | 0 | 0 | 0 | 1 | 0 | 1 |
| **8** | *Uroxys microcularis* Howden & Young, 1981 | 0 | 0 | 0 | 0 | 0 | 0 | 0 | 0 | 18 | 0 | 0 | 0 | 0 | 0 | 0 | 0 | 0 | 0 | 0 | 0 | 0 | 18 |
| **9** | *Uroxys micros* Bates, 1887 | 1 | 0 | 3 | 1 | 0 | 15 | 0 | 4 | 4 | 2 | 2 | 1 | 0 | 0 | 0 | 0 | 1 | 0 | 0 | 0 | 0 | 34 |
| **10** | *Uroxys platypyga* Howden & Young, 1981 | 0 | 0 | 0 | 0 | 0 | 0 | 0 | 5 | 1 | 0 | 0 | 0 | 0 | 0 | 0 | 0 | 0 | 0 | 0 | 0 | 0 | 6 |
|  | **Tribe Coprini Leach, 1815** |  |  |  |  |  |  |  |  |  |  |  |  |  |  |  |  |  |  |  |  |  |  |
| **11** | *Canthidium centrale* (Boucomont, 1928) | 0 | 0 | 0 | 0 | 0 | 0 | 0 | 4 | 10 | 2 | 0 | 3 | 0 | 0 | 0 | 0 | 0 | 0 | 0 | 2 | 0 | 21 |
| **12** | *Canthidium moroni* Kohlmann & Solis, 2006 | 0 | 0 | 0 | 0 | 0 | 0 | 0 | 3 | 0 | 0 | 0 | 0 | 0 | 0 | 0 | 0 | 0 | 0 | 0 | 0 | 0 | 3 |
| **13** | *Canthidium pseudoperceptibile* Kohlmann & Solis, 2006 | 1 | 0 | 0 | 0 | 0 | 0 | 1 | 2 | 0 | 0 | 0 | 2 | 1 | 0 | 0 | 0 | 0 | 0 | 0 | 0 | 0 | 7 |
| **14** | *Canthidium pseudopuncticolle* Solis & Kohlmann, 2004 | 0 | 0 | 0 | 0 | 0 | 0 | 0 | 0 | 0 | 0 | 0 | 0 | 6 | 0 | 0 | 26 | 0 | 13 | 0 | 2 | 2 | 49 |
| **15** | *Copris laeviceps* Harold, 1869 | 0 | 0 | 1 | 0 | 0 | 0 | 0 | 1 | 1 | 0 | 3 | 21 | 1 | 0 | 2 | 4 | 5 | 5 | 0 | 5 | 0 | 49 |
| **16** | *Copris lugubris* Boheman, 1858 | 0 | 0 | 1 | 0 | 0 | 0 | 2 | 0 | 2 | 1 | 1 | 21 | 8 | 1 | 15 | 110 | 5 | 332 | 0 | 4 | 34 | 537 |
| **17** | *Copris sallei* Harold, 1869 | 0 | 0 | 1 | 0 | 0 | 0 | 0 | 0 | 0 | 0 | 0 | 0 | 0 | 0 | 0 | 0 | 0 | 0 | 0 | 0 | 0 | 1 |
| **18** | *Ontherus mexicanus* Harold, 1868 | 404 | 99 | 535 | 108 | 8 | 3 | 0 | 0 | 0 | 0 | 0 | 0 | 0 | 0 | 0 | 0 | 0 | 0 | 0 | 0 | 0 | 1157 |
|  | **Tribe Dichotomiini Tarasov & Dimitrov, 2016** |  |  |  |  |  |  |  |  |  |  |  |  |  |  |  |  |  |  |  |  |  |  |
| **19** | *Dichotomius amplicollis* Harold, 1869 | 0 | 0 | 0 | 0 | 2 | 3 | 0 | 17 | 17 | 13 | 9 | 14 | 5 | 6 | 17 | 23 | 2 | 13 | 0 | 15 | 37 | 193 |
| **20** | *Dichotomius annae* Kohlmann & Solís, 1997 | 0 | 0 | 0 | 0 | 2 | 0 | 2 | 1 | 3 | 8 | 1 | 13 | 4 | 0 | 0 | 0 | 0 | 2 | 0 | 4 | 26 | 66 |
| **21** | *Dichotomius satanas* (Harold, 1867) | 0 | 0 | 16 | 1 | 1 | 0 | 0 | 1 | 0 | 0 | 9 | 7 | 0 | 0 | 0 | 0 | 3 | 0 | 0 | 0 | 0 | 38 |
|  | **Tribe Deltochilini Lacordaire, 1856** |  |  |  |  |  |  |  |  |  |  |  |  |  |  |  |  |  |  |  |  |  |  |
| **22** | *Canthon cyanellus* LeConte, 1859 | 0 | 0 | 0 | 0 | 0 | 0 | 0 | 0 | 2 | 0 | 1 | 6 | 42 | 0 | 6 | 32 | 3 | 179 | 0 | 4 | 9 | 284 |
| **23** | *Canthon eurycelis* Bates, 1887 | 0 | 0 | 1 | 0 | 0 | 0 | 0 | 2 | 0 | 1 | 2 | 2 | 0 | 0 | 0 | 0 | 0 | 0 | 0 | 0 | 0 | 8 |
| **24** | *Canthon femoralis* Chevrolat, 1834 | 0 | 0 | 0 | 0 | 0 | 0 | 0 | 0 | 0 | 0 | 0 | 0 | 0 | 0 | 0 | 12 | 0 | 64 | 0 | 0 | 3 | 79 |
| **25** | *Canthon indigaceus* LeConte, 1866 | 0 | 0 | 0 | 0 | 0 | 9 | 1 | 1 | 2 | 13 | 0 | 0 | 27 | 0 | 0 | 8 | 2 | 61 | 0 | 1 | 2 | 127 |
| **26** | Canthon leechi (Martínez, Halffter & Halffter, 1969) | 0 | 0 | 0 | 0 | 0 | 0 | 0 | 0 | 0 | 0 | 0 | 0 | 0 | 0 | 0 | 0 | 0 | 0 | 0 | 0 | 1 | 1 |
| **27** | *Canthon morsei* Howden, 1966 | 0 | 0 | 0 | 0 | 0 | 0 | 0 | 0 | 0 | 0 | 0 | 0 | 0 | 0 | 0 | 0 | **1** | 0 | 0 | 0 | 0 | 1 |
| **28** | *Canthon subhyalinus* Harold, 1867 | 5 | 0 | 2 | 0 | 0 | 0 | 0 | 0 | 0 | 0 | 0 | 0 | 0 | 0 | 0 | 0 | 0 | 0 | 0 | 0 | 0 | 7 |
| **29** | *Canthon vazquezae* (Martínez, Halffter & Halffter, 1964) | 909 | 28 | 106 | 6 | 167 | 124 | 0 | 69 | 93 | 9 | 212 | 64 | 3 | 0 | 1 | 0 | 0 | 0 | 0 | 3 | 0 | 1794 |
| **30** | *Cryptocanthon* aff. *brevisetosus* | 1 | 0 | 1 | 0 | 0 | 0 | 0 | 0 | 0 | 0 | 0 | 0 | 0 | 0 | 0 | 0 | 0 | 0 | 0 | 0 | 0 | 2 |
| **31** | *Deltochilum mexicanum* Burmeister, 1848 | 1016 | 97 | 791 | 188 | 54 | 24 | 0 | 60 | 33 | 0 | 46 | 14 | 0 | 0 | 0 | 0 | 0 | 0 | 0 | 0 | 0 | 2323 |
| **32** | *Deltochilum pseudoparile* Paulian, 1938 | 232 | 19 | 153 | 20 | 26 | 15 | 0 | 25 | 9 | 2 | 33 | 45 | 0 | 0 | 0 | 0 | 0 | 0 | 0 | 0 | 1 | 580 |
| **33** | *Deltochilum sublaeve* Bates 1887 | 6 | 0 | 7 | 0 | 1 | 1 | 0 | 2 | 1 | 0 | 25 | 27 | 1 | 0 | 0 | 0 | 1 | 2 | 0 | 0 | 2 | 76 |
| **34** | *Pseudocanthon perplexus* LeConte, 184 | 0 | 0 | 0 | 0 | 0 | 0 | 0 | 0 | 0 | 0 | 0 | 0 | 0 | 0 | 0 | 1 | 0 | 10 | 0 | 0 | 0 | 11 |
|  | **Tribe Oniticellini Kolbe, 1905** |  |  |  |  |  |  |  |  |  |  |  |  |  |  |  |  |  |  |  |  |  |  |
| **35** | *Euoniticellus intermedius* Reiche, 1849 | 0 | 0 | 0 | 0 | 0 | 0 | 0 | 0 | 0 | 0 | 0 | 0 | 0 | 0 | 0 | 1 | 0 | 0 | 0 | 0 | 0 | 1 |
| **36** | *Eurysternus angustulus* Harold, 1869 | 0 | 0 | 15 | 8 | 29 | 14 | 0 | 84 | 14 | 4 | 104 | 37 | 0 | 0 | 0 | 0 | 1 | 0 | 0 | 4 | 0 | 314 |
| **37** | *Eurysternus caribaeus* (Herbst, 1789) | 20 | 1 | 1 | 0 | 34 | 12 | 0 | 47 | 7 | 0 | 38 | 20 | 1 | 0 | 0 | 0 | 1 | 0 | 0 | 0 | 1 | 183 |
| **38** | *Eurysternus foedus* Guérin, 1844 | 0 | 0 | 1 | 0 | 0 | 0 | 0 | 2 | 0 | 0 | 2 | 1 | 0 | 0 | 0 | 2 | 0 | 0 | 0 | 0 | 0 | 8 |
| **39** | *Eurysternus magnus* Castelnau, 1840 | 17 | 2 | 1 | 4 | 2 | 1 | 0 | 1 | 1 | 3 | 0 | 0 | 0 | 0 | 0 | 0 | 0 | 0 | 0 | 0 | 0 | 32 |
| **40** | *Eurysternus maya* Génier, 2009 | 579 | 100 | 691 | 99 | 197 | 54 | 0 | 43 | 5 | 0 | 65 | 27 | 0 | 0 | 0 | 0 | 0 | 0 | 0 | 0 | 0 | 1860 |
| **41** | *Eurysternus mexicanus* Harold, 1869 | 0 | 0 | 1 | 0 | 1 | 3 | 1 | 1 | 10 | 5 | 1 | 65 | 4 | 0 | 2 | 34 | 1 | 129 | 0 | 11 | 6 | 275 |
|  | **Tribe Onthophagini Burmeister, 1846** |  |  |  |  |  |  |  |  |  |  |  |  |  |  |  |  |  |  |  |  |  |  |
| **42** | *Digitonthophagus gazella* (Fabricius, 1787) | 0 | 0 | 0 | 0 | 0 | 0 | 0 | 0 | 0 | 0 | 0 | 0 | 0 | 0 | 0 | 127 | 0 | 127 | 0 | 0 | 0 | 254 |
| **43** | *Onthophagus batesi* Howden & Cartwright, 1963 | 0 | 0 | 3 | 0 | 2 | 3 | 0 | 0 | 0 | 0 | 0 | 1 | 2 | 11 | 118 | 427 | 44 | 738 | 0 | 0 | 20 | 1369 |
| **44** | *Onthophagus corrosus* Bates, 1887 | 0 | 0 | 0 | 0 | 0 | 6 | 21 | 0 | 7 | 3 | 0 | 150 | 24 | 3 | 66 | 444 | 9 | 706 | 0 | 218 | 479 | 2136 |
| **45** | *Onthophagus crinitus* Harold, 1869 | 4 | 0 | 0 | 0 | 4 | 3 | 0 | 13 | 8 | 2 | 4 | 3 | 0 | 0 | 0 | 0 | 0 | 0 | 0 | 0 | 1 | 42 |
| **46** | *Onthophagus cyanellus* Bates, 1887 | 17 | 0 | 0 | 0 | 0 | 0 | 0 | 0 | 0 | 0 | 0 | 0 | 0 | 0 | 0 | 0 | 0 | 0 | 0 | 0 | 0 | 17 |
| **47** | *Onthophagus incensus* Say, 1835 | 12 | 0 | 13 | 2 | 87 | 49 | 26 | 32 | 6 | 2 | 49 | 71 | 3 | 0 | 3 | 5 | 2 | 1 | 0 | 11 | 8 | 382 |
| **48** | *Onthophagus landolti* Harold, 1880 | 0 | 0 | 0 | 0 | 0 | 0 | 2 | 1 | 5 | 8 | 0 | 6 | 8 | 0 | 33 | 46 | 9 | 202 | 0 | 100 | 131 | 551 |
| **49** | *Onthophagus maya* Zunino, 1981 | 0 | 0 | 0 | 0 | 0 | 0 | 0 | 0 | 0 | 0 | 0 | 0 | 0 | 0 | 1 | 0 | 0 | 0 | 0 | 0 | 0 | 1 |
| **50** | *Onthophagus veracruzensis* Delgado & Pensado, 1998 | 0 | 0 | 0 | 0 | 0 | 0 | 0 | 0 | 0 | 0 | 1 | 3 | 0 | 0 | 0 | 0 | 0 | 0 | 0 | 0 | 0 | 4 |
| **51** | *Onthophagus yucatanus* Delgado, Peraza & DeLoya, 2006 | 0 | 0 | 0 | 0 | 0 | 0 | 0 | 1 | 2 | 0 | 0 | 0 | 0 | 0 | 0 | 0 | 0 | 0 | 0 | 0 | 0 | 3 |
|  | **Tribe Phanaeini Hope, 1838** |  |  |  |  |  |  |  |  |  |  |  |  |  |  |  |  |  |  |  |  |  |  |
| **52** | *Coprophanaeus corythus* (Harold, 1863) | 22 | 1 | 20 | 3 | 3 | 0 | 3 | 2 | 3 | 1 | 20 | 34 | 3 | 0 | 10 | 27 | 2 | 44 | 6 | 1 | 65 | 270 |
| **53** | *Phanaeus endymion* Harold, 1863 | 0 | 0 | 0 | 0 | 0 | 0 | 0 | 1 | 3 | 1 | 2 | 1 | 0 | 0 | 3 | 9 | 0 | 5 | 1 | 0 | 6 | 32 |
| **54** | *Phanaeus sallei* Harold, 1863 | 2 | 0 | 0 | 0 | 1 | 0 | 0 | 1 | 3 | 1 | 3 | 9 | 1 | 0 | 0 | 2 | 0 | 2 | 0 | 10 | 7 | 42 |
| **55** | *Sulcophanaeus chryseicollis* (Harold, 1863) | 1 | 0 | 15 | 2 | 2 | 0 | 0 | 1 | 0 | 0 | 2 | 4 | 0 | 0 | 0 | 0 | 0 | 0 | 0 | 0 | 0 | 27 |
|  | **Σ n per land-cover class** | 3258 | 347 | 2382 | 442 | 630 | 353 | 59 | 460 | 273 | 83 | 644 | 723 | 146 | 22 | 278 | 1340 | 92 | 2635 | 7 | 422 | 861 | 15457 |
|  | **Σ n per window** | 3605 | | 2824 | | 1042 | | | 816 | | | 1513 | | | 1640 | | | 2727 | | 1290 | | |  |
|  | **Sample coverage land-cover class per window (%)** | 99 | 99 | 99 | 99 | 99 | 99 | 95 | 96 | 98 | 94 | 98 | 99 | 96 | 91 | 98 | 99 | 94 | 99 | 100 | 99 | 98 | 99 |
|  | **Sample coverage per window (%)** | 99 | | 99 | | 99 | | | 99 | | | 99 | | | 99 | | | 99 | | 99 | | |  |
