# Supplementary material for: Mechanisms of diversity maintenance in dung beetle assemblages in a heterogeneous tropical landscape
Source: PeerJ. 2020 Sep 8;8:e9860. doi: 10.7717/peerj.9860 (PMC7903913; doi:10.7717/peerj.9860)
Supplement: Supplemental Information 5 [file peerj-08-9860-s005.docx]

Species mean biomass.

|  | **Mean biomass (g)** | **(± S.E)** |
| --- | --- | --- |
| **Tribe Ateuchini Perty, 1830** |  |  |
| *Ateuchus candezei* (Harold, 1868) | 0.01210 |  |
| *Ateuchus perezvelai* Kohlmann, 2000 | N/A^a^ |  |
| *Ateuchus rodriguezi* Preudhomme de Borre, 1886 | 0.02116 | 0.0009 |
| *Bdelyropsis newtoni* Howden, 1971 | 0.00515 | 0.0004 |
| *Scatimus ovatus* Harold, 1862 | 0.00576 | 0.0057 |
| *Uroxys boneti* Pereira & Halffter, 1961 | 0.00190 | 0.0001 |
| *Uroxys deavilai* Delgado & Kohlmann, 2007 | N/A |  |
| *Uroxys microcularis* Howden & Young, 1981 | 0.00196 | 0.0002 |
| *Uroxys micros* Bates, 1887 | 0.00374 | 0.0006 |
| *Uroxys platypyga* Howden & Young, 1981 | 0.00383 | >0.001 |
| **Tribe Coprini Leach, 1815** |  |  |
| *Canthidium centrale* (Boucomont, 1928) | 0.05200 | 0.0245 |
| *Canthidium moroni* Kohlmann & Solis, 2006 | 0.00365 | 0.0005 |
| *Canthidium pseudoperceptibile* Kohlmann & Solis, 2006 | 0.00467 | 0.0008 |
| *Canthidium pseudopuncticolle* Solis & Kohlmann, 2004 | 0.00484 | 0.0005 |
| *Copris laeviceps* Harold, 1869 | 0.03107 | 0.0048 |
| *Copris lugubris* Boheman, 1858 | 0.09668 | 0.0112 |
| *Copris sallei* Harold, 1869 | 0.05600 |  |
| *Ontherus mexicanus* Harold, 1868 | 0.05402 | 0.0053 |
| **Tribe Dichotomiini Tarasov & Dimitrov, 2016** |  |  |
| *Dichotomius amplicollis* Harold, 1869 | 0.24788 | 0.0187 |
| *Dichotomius annae* Kohlmann & Solís, 1997 | 0.41015 | 0.0642 |
| *Dichotomius satanas* Harold, 1867 | 0.34456 | 0.0322 |
| **Tribe Deltochilini Lacordaire, 1856** |  |  |
| *Canthon cyanellus* LeConte, 1859 | 0.01807 | 0.0029 |
| *Canthon eurycelis* Bates, 1887 | 0.00580 | 0.0009 |
| *Canthon femoralis* Chevrolat, 1834 | 0.01305 | 0.0026 |
| *Canthon indigaceus* LeConte, 1866 | 0.04236 | 0.0026 |
| Canthon leechi (Martínez, Halffter & Halffter, 1969) | 0.00662 | 0.0006 |
| *Canthon morsei* Howden, 1966 | 0.01500 |  |
| *Canthon subhyalinus* Harold, 1867 | 0.00240 |  |
| *Canthon vazquezae* (Martínez, Halffter & Halffter, 1964) | 0.01723 | 0.0008 |
| *Cryptocanthon* aff. *brevisetosus* | 0.00180 |  |
| *Deltochilum mexicanum* Burmeister, 1848 | 0.34822 | 0.0302 |
| *Deltochilum pseudoparile* Paulian, 1938 | 0.06758 | 0.0033 |
| *Deltochilum sublaeve* Bates 1887 | 0.45730 | 0.0541 |
| *Pseudocanthon perplexus* LeConte, 184 | 0.00357 | 0.0002 |
| **Tribe Oniticellini Kolbe, 1905** |  |  |
| *Euoniticellus intermedius* Reiche, 1849 | N/A |  |
| *Eurysternus angustulus* Harold, 1869 | 0.01732 | 0.0008 |
| *Eurysternus caribaeus* (Herbst, 1789) | 0.06959 | 0.0080 |
| *Eurysternus foedus* Guérin, 1844 | 0.08363 | 0.0220 |
| *Eurysternus magnus* Castelnau, 1840 | 0.05454 | 0.0081 |
| *Eurysternus maya* Génier, 2009 | 0.07834 | 0.0045 |
| *Eurysternus mexicanus* Harold, 1869 | 0.03970 | 0.0034 |
| **Tribe Onthophagini Burmeister, 1846** |  |  |
| *Digitonthophagus gazella* (Fabricius, 1787) | 0.02342 | 0.0029 |
| *Onthophagus batesi* Howden & Cartwright, 1963 | 0.01467 | 0.0016 |
| *Onthophagus corrosus* Bates, 1887 | 0.00381 | 0.0002 |
| *Onthophagus crinitus* Harold, 1869 | 0.02530 | 0.0021 |
| *Onthophagus cyanellus* Bates, 1887 | 0.01849 | 0.0015 |
| *Onthophagus incensus* Say, 1835 | 0.01869 | 0.0018 |
| *Onthophagus landolti* Harold, 1880 | 0.00372 | 0.0004 |
| *Onthophagus maya* Zunino, 1981 | 0.01190 |  |
| *Onthophagus veracruzensis* Delgado & Pensado, 1998 | 0.00625 | 0.0019 |
| *Onthophagus yucatanus* Delgado, Peraza & DeLoya, 2006 | 0.00360 |  |
| **Tribe Phanaeini Hope, 1838** |  |  |
| *Coprophanaeus corythus* (Harold, 1863) | 0.66386 | 0.0525 |
| *Phanaeus endymion* Harold, 1863 | 0.13686 | 0.0104 |
| *Phanaeus sallei* Harold, 1863 | 0.17534 | 0.0180 |
| *Sulcophanaeus chryseicollis* Harold, 1863 | 0.20057 | 0.0149 |

^a^ NA: Insufficient individuals for estimating biomass.
