# Supplementary material for: Mechanisms of diversity maintenance in dung beetle assemblages in a heterogeneous tropical landscape
Source: PeerJ. 2020 Sep 8;8:e9860. doi: 10.7717/peerj.9860 (PMC7903913; doi:10.7717/peerj.9860)
Supplement: Supplemental Information 6 — F, Forest; SF, Second-growth forest; P, Pasture [file peerj-08-9860-s006.docx]

Tukey's Honest Significant Difference (HSD) results based on the generalized linear models (GLM) used to assess differences in abundance and biomass between windows and vegetation classes

Formula: log(abundance) ~ windows

Error distribution: Gaussian

| **Pairwise comparisons** | **Estimate** | **Std. Error** | **z value** | **Pr(>\|z\|)** |
| --- | --- | --- | --- | --- |
| W1-W2 | 0.263 | 0.708 | 0.372 | 1 |
| W1-W3 | -0.979 | 0.646 | -1.514 | 0.798 |
| W1-W4 | -1.139 | 0.646 | -1.762 | 0.644 |
| W1-W5 | -0.489 | 0.646 | -0.757 | 0.995 |
| W1-W6 | -0.502 | 0.646 | -0.776 | 0.994 |
| W1-W7 | -0.059 | 0.708 | -0.083 | 1 |
| W1-W8 | -0.938 | 0.646 | -1.452 | 0.831 |
| W2-W3 | -1.242 | 0.646 | -1.922 | 0.533 |
| W2-W4 | -1.402 | 0.646 | -2.170 | 0.368 |
| W2-W5 | -0.752 | 0.646 | -1.164 | 0.941 |
| W2-W6 | -0.765 | 0.646 | -1.184 | 0.936 |
| W2-W7 | -0.322 | 0.708 | -0.455 | 1 |
| W2-W8 | -1.202 | 0.646 | -1.860 | 0.576 |
| W3-W4 | -0.160 | 0.578 | -0.277 | 1 |
| W3-W5 | 0.490 | 0.578 | 0.847 | 0.99 |
| W3-W6 | 0.477 | 0.578 | 0.825 | 0.992 |
| W3-W7 | 0.920 | 0.646 | 1.424 | 0.845 |
| W3-W8 | 0.040 | 0.578 | 0.069 | 1 |
| W4-W5 | 0.650 | 0.578 | 1.124 | 0.951 |
| W4-W6 | 0.637 | 0.578 | 1.102 | 0.956 |
| W4-W7 | 1.080 | 0.646 | 1.671 | 0.704 |
| W4-W8 | 0.200 | 0.578 | 0.347 | 1 |
| W5-W6 | -0.013 | 0.578 | -0.022 | 1 |
| W5-W7 | 0.430 | 0.646 | 0.666 | 0.998 |
| W5-W8 | -0.449 | 0.578 | -0.778 | 0.994 |
| W6-W7 | 0.443 | 0.646 | 0.686 | 0.997 |
| W6-W8 | -0.437 | 0.578 | -0.756 | 0.995 |
| W7-W8 | -0.880 | 0.646 | -1.361 | 0.874 |

Formula: log(abundance) ~ vegetation classes. F: Forest; SF: Second-growth Forest; P: Pasture

Error distribution: Gaussian

| **Pairwise comparisons** | **Estimate** | **Std. Error** | **z value** | **Pr(>\|z\|)** |
| --- | --- | --- | --- | --- |
| F-P | -0.404 | 0.396 | -1.022 | 0.563 |
| F-SF | 0.335 | 0.368 | 0.908 | 0.635 |
| SF-P | 0.739 | 0.384 | 1.923 | 0.132 |

Formula: log(biomass) ~ windows

Error distribution: Gaussian

| **Pairwise comparisons** | **Estimate** | **Std. Error** | **z value** | **Pr(>\|z\|)** |
| --- | --- | --- | --- | --- |
| W1-W2 | -0.102 | 0.333 | -0.305 | 1.000 |
| W1-W3 | -1.400 | 0.304 | -4.603 | **< 0.001** |
| W1-W4 | -1.299 | 0.304 | -4.271 | **< 0.001** |
| W1-W5 | -0.925 | 0.304 | -3.042 | **0.048** |
| W1-W6 | -1.430 | 0.304 | -4.700 | **< 0.001** |
| W1-W7 | -1.190 | 0.333 | -3.571 | **0.008** |
| W1-W8 | -0.925 | 0.304 | -3.039 | **0.048** |
| W2-W3 | -1.299 | 0.304 | -4.269 | **< 0.001** |
| W2-W4 | -1.198 | 0.304 | -3.937 | **0.002** |
| W2-W5 | -0.824 | 0.304 | -2.708 | 0.119 |
| W2-W6 | -1.328 | 0.304 | -4.366 | **< 0.001** |
| W2-W7 | -1.088 | 0.333 | -3.266 | **0.024** |
| W2-W8 | -0.823 | 0.304 | -2.705 | 0.120 |
| W3-W4 | 0.101 | 0.272 | 0.372 | 1.000 |
| W3-W5 | 0.475 | 0.272 | 1.745 | 0.655 |
| W3-W6 | -0.030 | 0.272 | -0.109 | 1.000 |
| W3-W7 | 0.210 | 0.304 | 0.692 | 0.997 |
| W3-W8 | 0.476 | 0.272 | 1.749 | 0.653 |
| W4-W5 | 0.374 | 0.272 | 1.374 | 0.868 |
| W4-W6 | -0.131 | 0.272 | -0.480 | 1.000 |
| W4-W7 | 0.109 | 0.304 | 0.359 | 1.000 |
| W4-W8 | 0.375 | 0.272 | 1.377 | 0.867 |
| W5-W6 | -0.504 | 0.272 | -1.854 | 0.581 |
| W5-W7 | -0.265 | 0.304 | -0.870 | 0.989 |
| W5-W8 | 0.001 | 0.272 | 0.003 | 1.000 |
| W6-W7 | 0.240 | 0.304 | 0.789 | 0.994 |
| W6-W8 | 0.505 | 0.272 | 1.857 | 0.578 |
| W7-W8 | 0.265 | 0.304 | 0.873 | 0.988 |

Formula: Biomass(log) ~ Vegetation classes

Error distribution: Gaussian

| **Pairwise comparisons** | **Estimate** | **Std. Error** | **z value** | **Pr(>\|z\|)** |
| --- | --- | --- | --- | --- |
| F-P | -0.832 | 0.263 | -3.16 | **0.004** |
| F-SF | -0.189 | 0.245 | -0.771 | 0.720 |
| SF-P | 0.643 | 0.256 | 2.516 | **0.032** |
