# Supplementary figures and images for: Mechanisms of diversity maintenance in dung beetle assemblages in a heterogeneous tropical landscape
Source: PeerJ. 2020 Sep 8;8:e9860. doi: 10.7717/peerj.9860 (PMC7903913; doi:10.7717/peerj.9860)

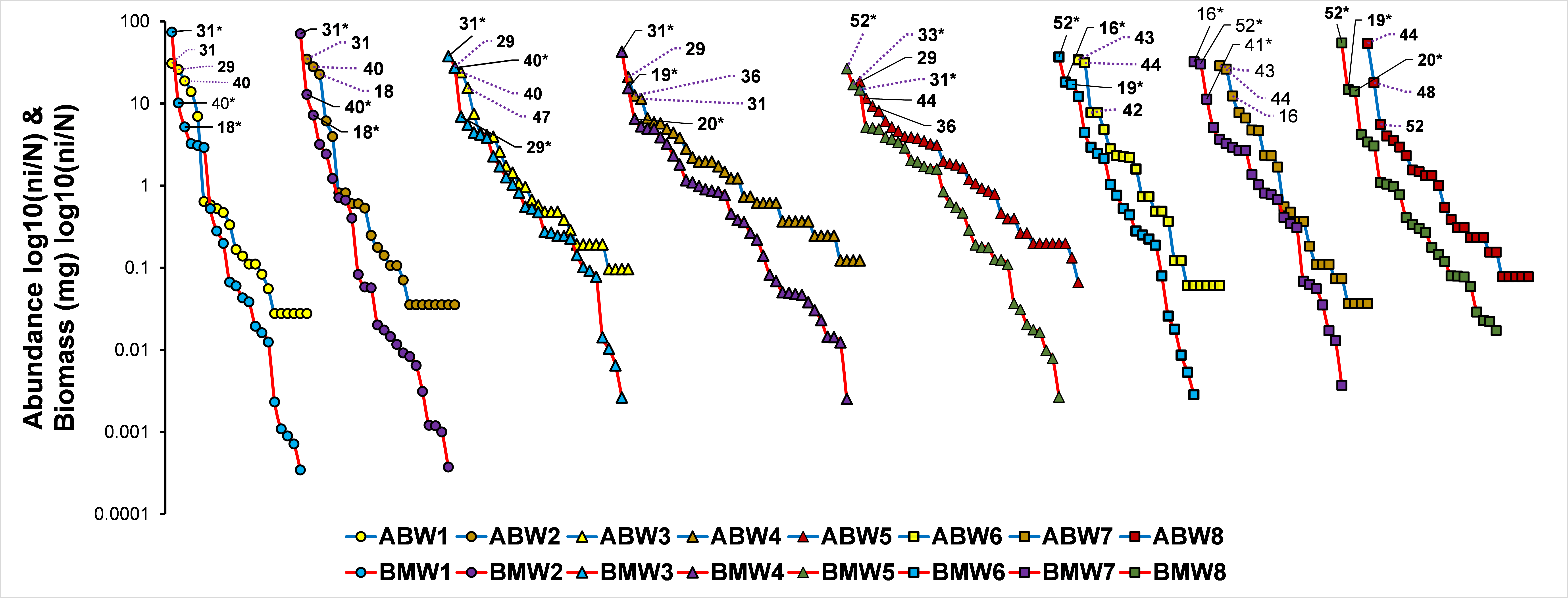

Supplement: Supplemental Information 7 — * symbols refer to species id within the rank-biomass curve. Species Id: 16 Copris lugubris; 18 Ontherus mexicanus; 19 Dichotomius amplicollis; 20 Dichotomius annae; 29 Canthon vazquezae; 31 Deltochilum mexicanum; 33 Deltochilum sublaeve; 36 Eurysternus angustulus; 40 Eurysternus maya; 43 Onthophagus batesi; 44 Onthophagus corrosus; 47 Onthophagus incensus; 48 Onthophagus landolti; 52 Coprophanaeus corythus. [file peerj-08-9860-s007.jpg]

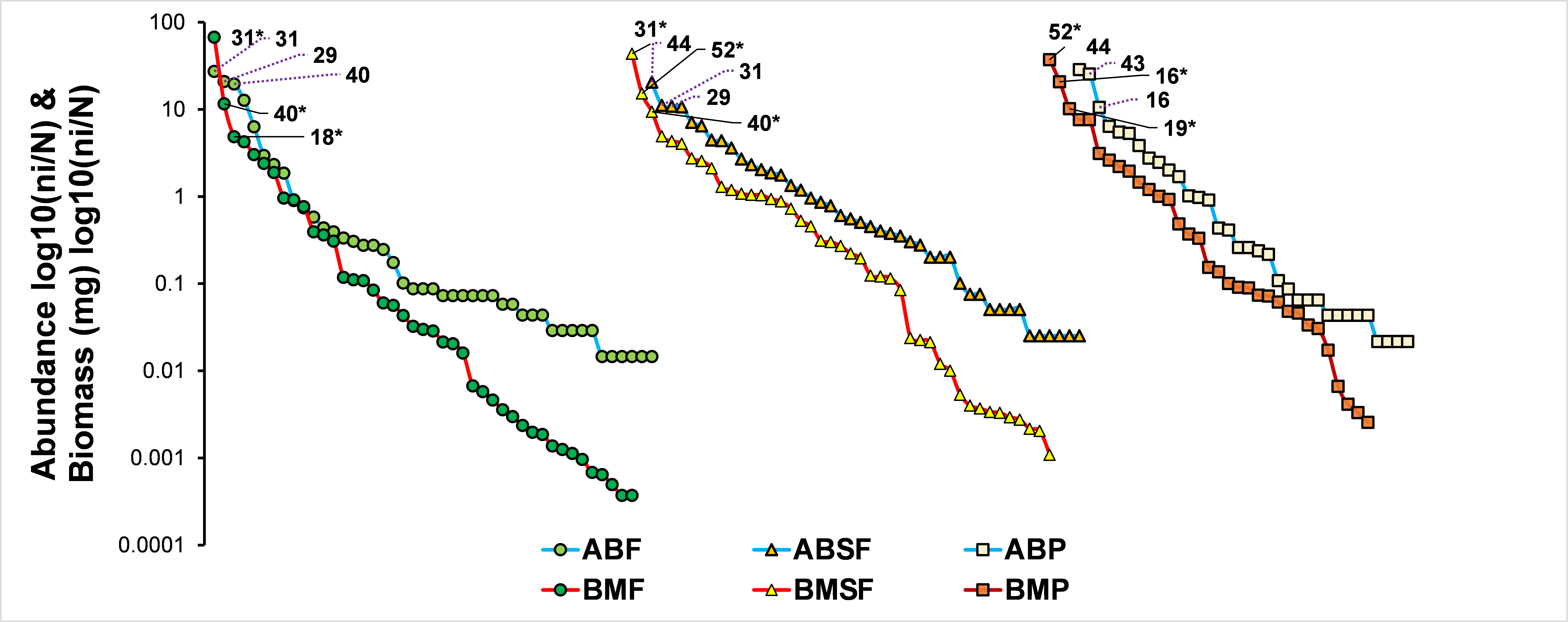

Supplement: Supplemental Information 8 — *symbols refer to species id within the rank-biomass curve. F: tropical forest, SF: second-growth forest; P: pasture. Species Id: 16 Copris lugubris; 18 Ontherus mexicanus; 19 Dichotomius amplicollis; 29 Canthon vazquezae; 31 Deltochilum mexicanum; 40 Eurysternus maya; 43 Onthophagus batesi; 44 Onthophagus corrosus; 52 Coprophanaeus corythus. [file peerj-08-9860-s008.jpg]
